# Isolation and identification of bioactive compounds in *Andrographis paniculata *(*Chuanxinlian*)

**DOI:** 10.1186/1749-8546-5-17

**Published:** 2010-05-13

**Authors:** Wen-Wan Chao, Bi-Fong Lin

**Affiliations:** 1 Department of Biochemical Science and Technology, College of Life Science, National Taiwan University, Taipei 10617, Taiwan

## Abstract

*Andrographis paniculata *(Burm. f.) Nees (Acanthaceae) is a medicinal plant used in many countries. Its major constituents are diterpenoids, flavonoids and polyphenols. Among the single compounds extracted from *A. paniculata*, andrographolide is the major one in terms of bioactive properties and abundance. Among the andrographolide analogues, 14-deoxy-11,12-didehydroandrographolide is immunostimulatory, anti-infective and anti-atherosclerotic; neoandrographolide is anti-inflammatory, anti-infective and anti-hepatotoxic; 14-deoxyandrographolide is immunomodulatory and anti-atherosclerotic. Among the less abundant compounds from *A. paniculata*, andrograpanin is both anti-inflammatory and anti-infective; 14-deoxy-14,15-dehydroandrographolide is anti-inflammatory; isoandrographolide, 3,19-isopropylideneandrographolide and 14-acetylandrographolide are tumor suppressive; arabinogalactan proteins are anti-hepatotoxic. The four flavonoids from *A. paniculata*, namely 7-*O*-methylwogonin, apigenin, onysilin and 3,4-dicaffeoylquinic acid are anti-atherosclerotic.

## Background

*Andrographis paniculata *(Burm. f.) Nees (Acanthaceae) (*A. paniculata*, *Chuanxinlian*), native to Taiwan, Mainland China and India, is a medicinal herb with an extremely bitter taste used to treat liver disorders, bowel complaints of children, colic pain, common cold and upper respiratory tract infection [1-3]. The aerial part of *A. paniculata *is commonly used in Chinese medicine. According to Chinese medicine theory, *A. paniculata *'cools' and relieves internal heat, inflammation and pain and is used for detoxication [4-6].

The herb contains diterpenoids, flavonoids and polyphenols as the major bioactive components [7,8]. This article reviews the constituents and pharmacological properties of *A. paniculata*, including its chemical components, biological activities and possible mechanisms. The literature search was conducted in Pubmed database (1984-2010), focused on language literature in English. The keywords used were selected from andrographolide, *A. paniculata *and its compounds with bioactivities. In comparison with other Chinese medicinal herbs, this well studied herb not only shows a wide variety of health benefits, but many bioactive compounds are also being identified. Furthermore, several derivatives have been semi-synthesized to enhance their bioactivity than original compounds, suggesting a potential for drug development. The authors read more than 200 full articles and a total of 124 peer-reviewed papers focused on anti-inflammation, anti-cancer, immunomodulation, anti-infection, anti-hepatotoxicity, anti-atherosclerosis, anti-diabetes and anti-oxidation were selected for this review.

### Bioactive constituents

**Figure 1:**
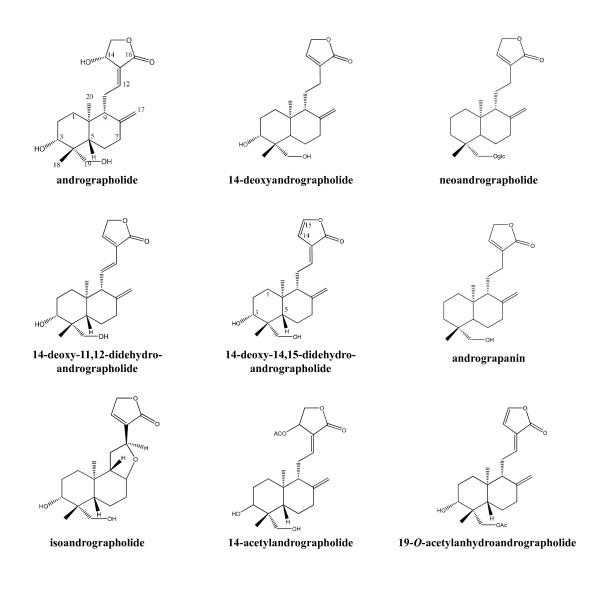
**Structures and bioactivities of compounds isolated from *A. paniculata***.

**Figure 2:**
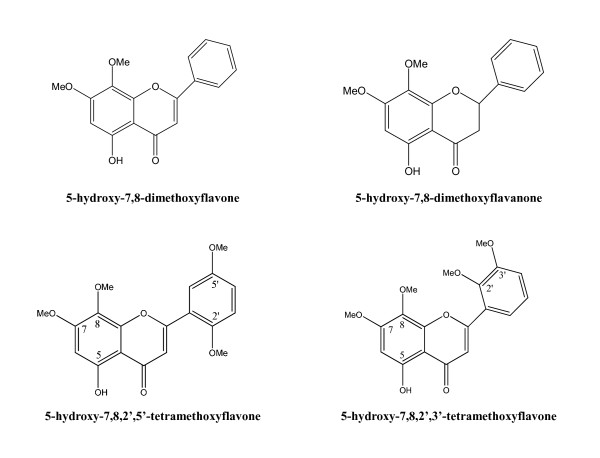
**Structures and bioactivities of flavonoids isolated from *A. paniculata***.

Active compounds extracted with ethanol or methanol from the whole plant, leaf and stem [9-11] include over 20 diterpenoids and over ten flavonoids have been reported from *A. paniculata *[12,13]. Andrographolide (C_20_H_30_O_5_) is the major diterpenoid in *A. paniculata*, making up about 4%, 0.8~1.2% and 0.5~6% in dried whole plant, stem and leaf extracts respectively [9,11,14]. The other main diterpenoids are deoxyandrographolide, neoandrographolide, 14-deoxy-11,12-didehydroandrographide and isoandrographolide [9,15] (Table 1, Figure 1). From ethyl acetate (EtOA_C_)-soluble fraction of the ethanol or methanol extract, 5-hydroxy-7,8-dimethoxyflavone, 5-hydroxy-7,8,2',5'-tetramethoxyflavone, 5-hydroxy-7,8,2',3'-tetramethoxyflavone, 5-hydroxy-7,8,2'-trimethoxyflavone, 7-*O*-methylwogonin and 2'-methyl ether were isolated as the main flavonoids [15-18] (Figure 2).

**Table 1:** Bioactivities of compounds isolated from *A. paniculata*

Names	Bioactivities	References
Andrographolide	Bioactivities	
14-deoxyandrographolide	↑ activation of NOS and guanylate cyclase ↑ vasorelaxation *in vitro *and *in vivo*	[102,103,106]
neoandrographolide	↓ NO, PGE_2_, iNOS and COX-2 in activated macrophages ↓ CCl_4_, tBHP-induced hepatotoxicity (*i.p *100 mg/kg, 3d)	[34,35,91]
14-deoxy-11,12-didehydroandrographolide	↑ muscle relexation. ↑ NO release from endothelial cells	[107,105]
14-deoxy-14,15-didehydroandrographolide	↑ cytotoxic activity and cell cycle arrest of tumor cells ↓ NF-κB-dependent trans-activation	[42,17]
andrograpanin	↑ protein kinase or p38 MAPKs pathways ↑ chemokine SDF-1α induced chemotaxis in Jurkat and THP-1 cells	[37,87]
isoandrographolide	↑ cell-differentiation-inducing activity ↓ proliferation of HL-60 cells	[10,44]
14-acetylandrographolide	↓ growth of leukeamia, ovarian, renal cancer cells	[47]
19-*O*-acetylanhydroandrographolide	↓ NF-κB-dependent trans-activation	[17]

Andrographolide exhibits multiple pharmacological properties and is a potential chemotherapeutic agent [19]. Andrographolide contains an α-alkylidene γ-butyrolactone moiety and three hydroxyls at C-3, C-19 and C-14 responsible for the cytotoxic activities of andrographolide against many cancer cell lines [19]. Andrographolide is abundant in leaves and can be easily isolated from the crude plant extracts as crystalline solid [5,10,17,20,21].

### Pharmacological properties

*A. paniculata *exhibits a vast range of pharmacological properties (Tables 2 and 3).

**Table 2:** Pharmacological properties of various extracts of *A. paniculata*

Chemicals	Pharmacological properties	References
methanol extract	restore plasma lipid peroxidation, ALT, AST activities in CCl_4_-treated rats (orally 1 g/kg BW, 14d)	[94]
ethanol extract	↑ serum anti-*Salmonella typhinurium *IgG levels ↑ IFN-γ in Con A-stimulated splenocytes of mice (orally, 25 or 50 mg/kg BW, 14d)	[76]
	↑ antibody and the delayed-type hypersensitivity response (orally 25 mg/kg, 7d)	[74]
	↑ G0/G1 phase ↑ mitochondrial CYP and expression of Bax in human leukemic HL-60 cells	[49]
	↓ expression of EBV lytic proteins during the viral lytic cycle in P3HR1 cells	[82]
	↓ fasting serum glucose in diabetic rats (orally 0.1, 0.2, and 0.4 g/BW, 14d) ↓ liver and kidney TBARS levels ↑ liver GSH concentrations (orally 400 mg/kg BW, 14d)	[113]
95% ethanol extract	↓ RANTES secretion by human bronchial epithelial cells infected with influenza A virus H1N1	[86]
80% ethanol extract	↑ hepatic GPX, GR, CAT, SOD; ↓ lipid peroxidation (orally 50, 100 mg/kg BW, 14d)	[121]
70% ethanol extract	↑ CTL production through enhanced secretion of IL-2 and IFNγ by EL-4 T cells	[43]
	↓ serum NO, VEGF and TIMP-1, angiogenesis in melanoma cell implanted mice (*i.p*. 10 mg/d, 5d)	[56]
95% ethanol or EtOAc extract	↑ pi class of glutathione S-transferase expression in rat primary hepatocytes	[99]
EtOAc extract	↓ NF-κB trans-activation assayed by NF-κB-dependent luciferase activity ↓ *ex-vivo *NO and PGE_2 _production by LPS/IFN-γ-stimulated peritoneal macrophages ↓ LPS-induced acute inflammation in mice (orally 0.78~3.12 mg/kg BW, 7d)	[5,39]
aqueous extract	↑ protect nicotine-induced toxicity in brain (*i.p*. 250 mg/kg BW, 7d) ↓ nicotine induced DNA fragmentation in lymphocytes, lipid peroxidation, protein oxidation	[93,92]
	↓ systolic blood pressure of SHR and WKY rats (*i.p*. 0.7, 1.4, 2.8 g/kg BW)	[101]
	↓ blood glucose in STZ-induced hyperglycaemic rats (50 mg/kg BW, 10d)	[115]
	↑ hepatic CAT, SOD and GST activities in lymphoma bearing mice (orally 10~30 mg/d)	[123]

d: day; BW: body weight

**Table 3:** Pharmacological properties of andrographolide

Pharmacological properties	References
***Anti-inflammation***	
↓ LPS-induced NO production by suppressing iNOS	[27]
↓ complement 5a-induced macrophage recruitment *via *↓ ERK1/2 and PI3K signal pathways	[30]
↓ binding of NF-κB oligonucleotide to nuclear proteins *via *↓ERK1/2 or PI3/AKt signal pathway	[28,31-33]
	
***Anti-cancer***	
↓ proliferation of HL-60 cells, the JAK-STAT3 pathway	[44,63]
↑ caspase 8 dependent Bid cleavage, caspase 3, 9 activity, TRAIL-induced apoptosis, cell cycle arrest	[48,52,53,63]
↑ tumor suppressor p53 expression, MAPKs (p38 kinase, JNK, ERK1/2) signaling pathway	[50,54]
↓ oncogene v-Src protein expression and v-Src-induced transformation	[55]
↓ E-selectin expression on endothelial cells for cancer cells adhension, MMP-7 expression in cancer cells	[57,58]
↓ tumor in melanoma subcutaneously implanted mice (orally 200, 400 mg/kg BW, 10d)	[45]
	
***Immunomodulation***	
↑ proliferation and IL-2 induction in hPBL	[31]
↑ antibody and the delayed-type hypersensitivity response (orally 1 mg/kg, 7d)	[74]
↑ serum anti-*Salmonella *IgG, IFN-γ in activated splenocytes of mice (orally 1, 4 mg/kg BW, 14d)	[76]
↓ TNF-α and GM-CSF in BALF of OVA-sensitized and nasally-challenged mice (*i.p*. 3~30 mg/kg BW)	[72]
↓ IL-4, IL-5 and IL-13 in BALF and OVA-specific IgE in serum of OVA-sensitized mice (*i.p*. 0.~ 1 mg/kg BW, twice)	[71]
↓ NF-κB expression in lung and airway epithelial cells ↓ infiltration of inflammatory cells in lung, airway hyperreactivity (*i.p*. 2 μg/g BW, 7d)	[69]
↓ expression of IL-2 via ↓ NFAT and ↑ JNK phosphorylation in murine T-cells	[67]
↓ LPS induced dopaminergic neurodegeneration in primary rat mesencephalic neuron-glial cultures	[70]
↓ IL-2 production, proliferation, antibody production, T cell activation in EAE (*i.p*. 4 mg/kg BW)	[68]
↓ symptom and immunological markers in patients with RA (30% andrographolide tablet, 14 weeks)	[73]
	
***Anti-infection***	
↓ HIV induced cell cycle dysregulation, ↑ CD4^+ ^lymphocyte levels in HIV-1 infected individuals	[79,80]
↑ viricidal activity against HSV-1, EBV, *via *↓ producing mature virus particle	[81,82]
	
***Anti-hepatotoxicity***	
↑ CYP1A1 and CYP1A2 mRNA in mouse hepatocytes, synergistic effect in with a CYP1A1 inducer	[95,96]
	
↑ expression of the pi class of glutathione S-transferase	[99]
↓ CCl_4_, tBHP-induced hepatotoxicity (*i.p *100 mg/kg, 3d)	[91]
	
***Anti-atherosclerosis***	
↑ HUVECs apoptosis *via *enhancement of PI3K-Akt activity	[108]
↓ thrombin-induced platelet aggregation *via *↓ ERK1/2 pathway	[109]
	
***Anti-hyperglycemic effect***	
↓ plasma glucose concentrations of STZ-diabetic rats (oral 1.5 mg/Kg) ↑ mRNA and protein levels of GLUT4 in soleus muscle	[117,118]
	
***Anti-Oxidation***	
↓ MDA formation	[91]
↑ GSH, SOD activity	[92,93]

#### Anti-inflammation effects

Systemic inflammation was suggested to be associated with increased risk of chronic diseases such as cardio-vascular disease, cancer and insulin resistance [22]. Inflammation involves macrophage and T lymphocyte activation as well as the release of pro-inflammatory mediators, such as tumour necrosis factor (TNF)-α, interleukin (IL)-1, IL-6, interferon (IFN)-γ, nitric oxide (NO) and cell adhesion molecules which in turn amplify the inflammation [23]. Effective modulation of the aberrant production of these molecules may reduce inflammation [24,25].

A previous study demonstrated that intraperitoneal (*i.p*.) administration of *A. paniculata *methanol extract for five consecutive days (50 mg/day) inhibited 65% NO production by peritoneal macrophage and significantly inhibited carageenan induced paw oedema formation in mice [26]. Andrographolide inhibits nitric oxide (NO) production and the expression and stability of inducible synthase (iNOS) protein in lipopolysaccharide (LPS)-stimulated RAW264.7 (RAW) cells [27,28]. Andrographolide inhibits oxygen radical production in neutrophils [29], inhibits macrophage migration [30], NF-κB activity [31,32] as well as TNF-α and IL-12 production [33]. These anti-inflammatory activities of andrographolide may be a result of its interference with protein kinase C-dependent pathway, extracellular signal-regulated kinase1/2 (ERK1/2) or PI3K/Akt signalling pathway.

Neoandrographolide, isolated from EtOAc portion in methanol extract, suppresses NO production both *in vitro *and *ex vivo *in bacillus Calmette-Guéin (BCG)-induced peritoneal macrophages [34] in mice. Neoandrographolide inhibits *in vitro *TNFα and PGE_2 _production in RAW cells, suppresses ear oedema induced by dimethyl benzene in mice [35,36]. Andrograpanin, a hydrolysate from neoandrographolide, reduces NO, TNFα and IL-6 production in LPS-activated macrophage cells derived from bone marrow in mice, possibly due to down-regulation of p38 mitogen-activated protein kinase (MAPKs) signalling pathways [37].

To screen for anti-inflammatory herbs, we transfected luciferase (with NF-κB binding site) into murine macrophage RAW cells and measured the suppression of luciferase activities [38]. EtOAc extract of *A. paniculata *inhibited NF-κB-dependent luciferase gene expression and suppressed TNF-α, IL-6, macrophage inflammatory protein-2 (MIP-2), NO and PGE_2 _production by LPS/IFNγ-stimulated RAW cells [5,39]. In an endotoxin shock model, the mice oral supplemented with AP EtOAc extract had significantly lower TNF-α, MIP-2, IL-12 or NO in serum or peritoneal macrophages when challenged with LPS. Those LPS-challenged mice also had lower infiltration of inflammatory cells into the lung and higher survival rate [39].

**Figure 3:**
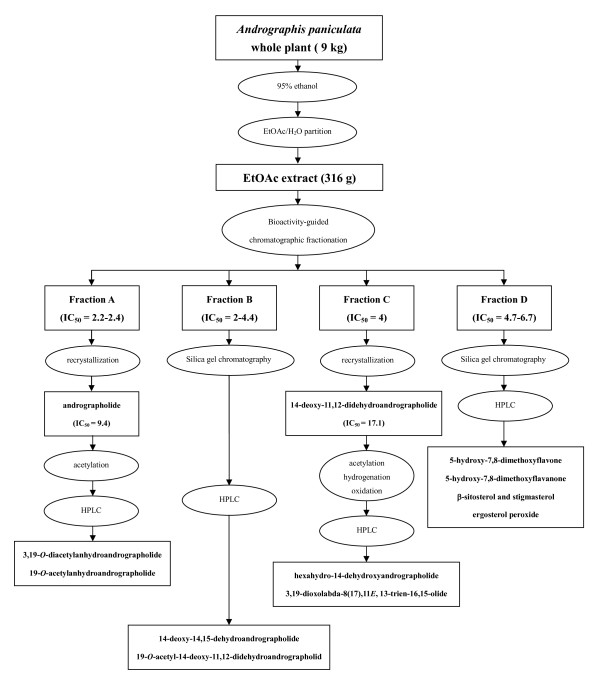
**Extraction procedure for the isolation and identification *A. paniculata *pure compounds from EtOAc extract**. Dried whole plant of *A. paniculata *is pre-extracted with 95% ethanol and then partitioned in EtOAc/H_2_O for further fractionation.

**Figure 4:**
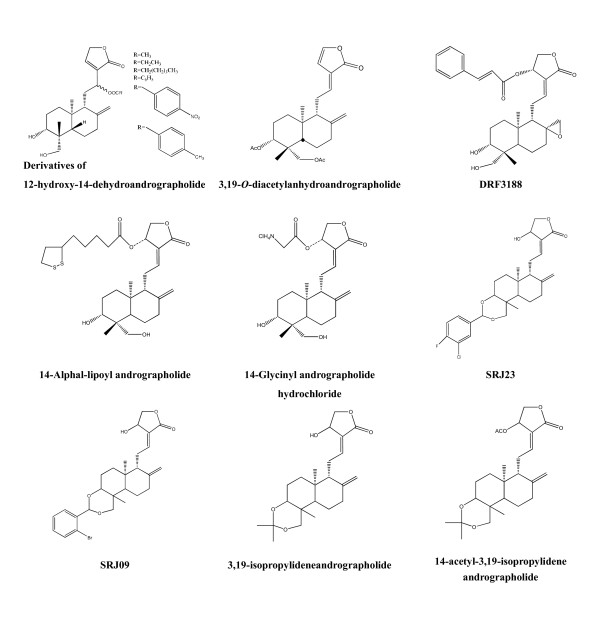
**Structures and bioactivities of synthesized analogues**.

Using bioactivity-guided chromatographic separation, we isolated the anti-inflammatory compounds from the EtOAc extract of *A. paniculata *and identified eight compounds with anti-inflammatory properties [17], namely andrographolide, 14-deoxy-11,12-didehydroandrographolide, ergosterol peroxide, 14-deoxy-14,15-dehydroandrographolide, 5-hydroxy-7,8-dimethoxyflavone, 19-*O*-acetyl-14-deoxy-11,12-didehydroandrographolide, β-sitosterol, stigmasterol and 5-hydroxy-7,8-dimethoxyflavanone (Figure 3). The IC_50 _values of each compound for the inhibition of the pro-inflammatory cytokines were similar to those for NF-κB transcriptional activation (Table 4). Acetylation of andrographolide yields two compounds, namely 3,19-*O*-diacetylanhydroandrographolide and 19-*O*-acetylanhydroandrographolide. Other studies demonstrated that synthetic andrographolide derivatives such as 12-hydroxy-14-dehydroandrographolide derivatives and isopropylideneandrographolide had more inhibitory activities than andrographolide [13,40] (Table 5, Figure 4). Therefore, the NF-κB dependent luciferase reporter assay may help screen anti-inflammatory Chinese medicinal herbs and isolate their bioactive compounds [5].

**Table 4:** The IC_50 _values of NK-B transactivation and pro-inflammatory mediators of the compounds isolated or semi-synthesized from *A. paniculata *EtOAc extract

Compounds	NF-κB	TNFα	IL-6	MIP-2	NO
**Semi-synthetic analogues**			(μg/ml)		
3,19-*O*-diacetylanhydroandrographolide	2.2	2.89	2.08	1.29	2.02
19-*O*-acetylanhydroandrographolide	2.4	3.85	2.75	2.29	2.08
**Diterpenoids**					
14-deoxy-14,15-dehydroandrographolide	2.0	2.18	2.14	2.07	2.05
19-*O*-acetyl-14-deoxy-11,12-didehydroandrographolide	4.4	5.07	3.94	4.41	4.11
**Synthetic analogues**					
hexahydro-14-dehydroxyandrographolide	4.2	5.2	3.78	4.52	4.24
3,19-dioxolabda-8(17),11*E*, 13-trien-16,15-olide	4.1	5.78	3.97	4.67	4.52
**Steroids**					
ergosterol peroxide	4.7	4.71	5.32	4.55	4.67
β-sitosterol and stigmasterol	5.2	5.34	5.97	4.88	4.55
**Flavonoids**					
5-hydroxy-7,8-dimethoxyflavone	6.1	4.33	5.34	3.63	5.11
5-hydroxy-7,8-dimethoxyflavanone	6.7	4.37	5.14	4.01	4.57
**Diterpenoids**					
andrographolide (major compound)	9.4	5.62	8.93	5.48	8.48
14-deoxy-11,12-didehydroandrographolide	17.1	20.64	23.6	15.03	11.26

NF-κB trans-activation activity was assay as following: RAW 264.7 macrophages transiently transfected with NF-κB reporter plasmids were pretreated various compounds and then stimulated with LPS 100 ng/mL/IFN-γ 1000 units/mL for further was estimated by the Dual-Glo Luciferase reporter assay. The collected cell supernatants were assayed for TNFα, IL-6, and MIP-2 productions using commercial ELISA kits. NO was determined by Griess assay [17].

**Table 5:** Bioactivities of synthetic analogues of andrographolide

Name	Bioactivities	References
Derivatives of 12-hydroxy-14-dehydroandrographolide	↓ TNF-α and IL-6 secretion in mouse macrophages	[13]
3,19-*O*-diacetylanhydroandrographolide	↓ NF-κB-dependent trans-activation in the RAW264.7 cells	[17]
DRF3188	block MCF-7 cell cycle at the G0/G1 phase ↑ cell cycle inhibitor, p27 ↓ the levels of CDK4	[46]
14-Alphal-lipoyl andrographolide	↑ against H9N2, H5N1 and H1N1 viruses to reduced the death rate, prolonged life and inhibited lung consolidation and viral titers	[83]
14-Glycinyl andrographolide hydrochloride	↓ reduced virulence factor production	[84]
SRJ23	↑ G1 arrest and apoptosis in MCF-7 and HCT-116	[62]
SRJ09	↑ G1 arrest and apoptosis in MCF-7 and HCT-116	[62]
3,19-isopropylideneandrographolide	↑cyotoxicity against MCF-7 and HCT-116	[47]
14-acetyl-3,19-isopropylidene andrographolide	↑cyotoxicity against MCF-7 and HCT-116	[47]

#### Anti-cancer effects

Kumar *et al*. fractionated the methanol extract of *A. paniculata *into dichloromethane, petroleum ether and aqueous extracts and found that only the dichloromethane fraction significantly inhibited the proliferation of HT-29 colon cancer cells [41]. They further fractionated the dichloromethane extract and yielded three diterpene compounds, namely andrographolide, 14-deoxyandrographolide and 14-deoxy-11,12-didehydroandrographolide. Andrographolide showed the greatest anti-cancer activity on a range of cancer cells [41]. The *A. paniculata *ethanol extract showed cytotoxic activities against human cancer cell lines, such as Jurkat (lymphocytic), PC-3 (prostate), HepG2 (hepatoma) and Colon 205 (colonic) cells [42]. An *in vivo *study demonstrated that *A. paniculata *70% ethanol extract and andrographlide increased the life spans of mice injected with thymoma cells [43]. Isolated from 85% ethanol extract of *A. paniculata*, andrographolide and isoandrographolide exhibited higher antiproliferative activities in human leukaemia HL-60 cells than other 16 *ent*-labdane diterpenoids with IC_50_'s of 9.33 and 6.30 *μ*M respectively [44].

The anti-cancer mechanisms of andrographolide have been investigated [19]. Andrographolide and its analogues exert direct anti-cancer activities on cancer cells by cell-cycle arrest at G0/G1 phase through induction of cell-cycle inhibitory protein and decreased expression of cyclin-dependent kinase [45-49]. Other compounds may block the cell cycle progression at G2/M phase [42]. Andrographolide inhibits human hepatoma cell growth through activating c-Jun N-terminal kinase [50] or inducing cell differentiation [51]. Andrographolide induces apoptosis in human cancer cells via the activation of caspase 8, pro-apoptotic Bcl-2 family members Bax conformational change, release of cytochrome C from mitochondria and activation of caspase cascade [52] and/or via the activation of tumour suppressor p53 by ROS-dependent c-Jun NH_2_-terminal kinase (JNK) activation, thereby increasing p53 phosphorylation and protein stabilization [53,54]. Andrographolide may suppress an oncogene v-Src-induced transformation and down-regulate v-Src protein expression via the attenuation of ERK1/2 signalling pathway [55].

In addition, enhancement of immunity and inhibition of angiogenesis and tumour cell migration may also contribute to the anti-cancer effects. Inhibiting human cancer cell growth, *A. paniculata *extract enhances proliferation and IL-2 induction in human peripheral blood lymphocytes [41]. Sheeja *et al*. showed that the *A. paniculata *ethanol extract and andrographolide stimulated the cytotoxic T lymphocytes (CTL) activity through enhanced release of IL-2 and IFNγ in serum thereby inhibiting tumour growth [43]. The *A. paniculata *ethanol extract and andrographolide successfully inhibited the tumour specific capillary sprouting without damaging the pre-existing vasculature in mice injected with melanoma cells. *A. paniculata *extract inhibits tumour specific angiogenesis by down-regulating various proangiogenic molecules such as vascular endothelial growth factor (VEGF), NO and proinflammatory cytokines and up-regulating anti-angiogenic molecules such as IL-2 and tissue inhibitors of metalloproteinase-1 (TIMP-1) which prevent tumour metastasis [56]. As tumour cells can express high levels of sialyl Lewis surface antigens that interact with adhesion molecules E- and P-selectins on activated endothelial cells, cancer cell adhesion to endothelial cells followed by tumour extravasation results in metastasis. Andrographolide inhibits the adhesion of cancer cells to the activated endothelium by blocking E-selectin expression [57]. Andrographolide may also inhibit angiogenesis for tumour metastasis via down-regulating matrix metalloproteinases-7 (MMP-7) expression, possibly by inactivating activator protein-1 (AP-1) through suppressing PI3K/Akt signalling pathway [58,59].

A novel semi-synthetic analogue of andrographolide, DRF3188, shows anti-cancer activities at a lower dosage than andrographolide through a similar mechanism [46]. Synthesis and structure-activity relationships of andrographolide analogues as novel cytotoxic agents reveals that intact α-alkylidene γ-butyrolactone moiety of andrographolide, the D12(13) double bond, the C-14 hydroxyl or its ester moiety and the D8(17) double bond or epoxy moiety are responsible for the cytotoxic activities exhibited by andrographolide and its analogues [60]. Anti-cancer agents usually possess selective growth inhibition or cytotoxic properties [61]. The semi-synthesized andrographolide derivatives were screened against a panel of 60 human cancer cell lines. The results showed that 3,19-isopropylideneandrographolide was selective towards leukaemia and colon cancer cells whereas 14-acetylandrographolide was selective towards leukaemia, ovarian and renal cancer cells [47]. The benzylidene derivatives of andrographolide showed more potent anti-cancer activities than andrographolide [62]. The addition of andrographolide to 5-Fluorouracil induces synergistic apoptosis [54]. Moreover, andrographolide enhances the sensitivity of cancer cells to a chemotherapeutic drug, namely doxorubicin, mainly via suppressing JAK-STAT3 [63]. The results of these studies suggest a potential therapeutic strategy of combining andrographolide with chemotherapeutic agents to treat cancer.

#### Immunomodulatory effects

Immune responses such as proliferation of lymphocytes, antibody production and cytokines secretion are regulated under normal conditions. Every immunocompetent cell is controlled by other cells with antagonistic action [64]. The balance between type 1 T helper cell-mediated and type 2 Th cell-mediated immune responses is critical for immunoregulation.

*A. paniculata *dichloromethane extract significantly augments the proliferation of human peripheral blood lymphocytes (hPBL) at low concentrations [41]. The three diterpene compounds including andrographolide enhance proliferation and IL-2 secretion in hPBL [41]. Andrographolide enhances secretion of IL-2 and IFNγ by T cells and stimulates the production of cytotoxic T lymphocytes [43,65].

On the other hand, when murine T cell is stimulated with mitogen, IL-2 was decreased by andrographolide [66] possibly via reducing nuclear factor of activated T cells (NFAT) activities and increasing JNK phosphorylation [67]. Similarly, andrographolide interferes with T cell activation and reduces the severity of experimental autoimmune encephalomyelitis (EAE) in mice. Clinical signs of disease such as abnormal gait and limb paralysis, proliferation and IL-2 secretion of lymph node cells, as well as cell-dependent antibody production in EAE mice were reduced by andrographolide treatment [68]. Andrographolide is beneficial for inflammation-related immune dysregulatory diseases, such as allergic asthma, rheumatoid (RA) and neurodegenerative diseases via inhibition of the NF-κB signalling pathway [69]. Andrographolide reduces inflammation-mediated dopaminergic neurodegeneration in mesencephalic neuron-glial cultures by inhibiting microglial activation and production of proinflammatory factors such as TNFα, NO and PGE_2 _[70]. Andrographolide inhibits OVA-induced increases in total cells, eosinophils and IL-4, IL-5 and IL-13 levels in bronchoalveolar lavage fluid (BALF), and reduces serum level of OVA-specific IgE [71]. Andrographolide attenuated OVA-induced lung tissue eosinophils and airway mucus production, mRNA expression of E-selectin, chitinases, mucin Muc5ac and iNOS in lung tissues and airway hyperresponsiveness [71]. Andrographolide inhibits OVA-induced increases TNF-α and GM-CSF in BALF of OVA-sensitized and nasally-challenged mice [72]. A recent clinical study showed that *A. paniculata *extract (30% andrographolide) reduced the symptoms and certain immunological parameters such as serum immunoglobulins and complement components in patients with rheumatoid arthritis during a 14-week treatment [73].

Oral administration of the *A. paniculata *ethanol extract or andrographolide to mice stimulated antibody production and the delayed-type hypersensitivity response to sheep red blood cells [74]. Andrographolide increases spontaneous IFNγ and mitogen-stimulated TNF-α secretion by cultivated human peripheral blood cells [75]. Oral pre-treatment of the *A. paniculata *ethanol extract or andrographolide in mice immunized with an inactivated *Salmonella typhimurium *vaccine enhances *Salmonella*-specific IgG antibody and IFN-γ production [76]. Recent study demonstrated that the cyclophosphamide-potentiated DTH reaction was reversed by a pure powder mixture of andrographolide plus 14-deoxyandrographolide and 14-deoxy-11,12-didehydroandrographolide together. The mixture stimulated phagocytosis, and elevated antibody titer and plaque-forming cells in the spleen cells in mice [77].

#### Anti-infective effects

The aqueous extract of *A. paniculata *against anti-human immunodeficiency virus (HIV) was ruled out by testing the inhibitory activities against HIV in the H9 cell line [78]. Reddy *et al*. tested the anti-HIV activity of the n-hexane and methanol extracts of *A. paniculata*. Seven compounds, namely andrographolide, bis-andrographolide 14-deoxy-11,12-didehydroandrographolide, andrograpanin, 14-deoxyandrographolide, (±)-5-hydroxy-7,8-dimethoxyflavanone and 5-hydroxy-7,8-dimethoxyflavone. Andrographolide and 14-deoxy-11,12-didehydroandrographolide showed anti-HIV activity with 50% effective concentration (EC_50_) of 49 and 57 μg/ml respectively [79]. A phase I dose-escalating clinical trial of andrographolide in HIV positive patients reported a significant rise in the mean CD4^+ ^lymphocyte level of HIV patients. Andrographolide inhibits HIV-induced cell cycle dysregulation, leading to a rise in CD4^+ ^lymphocyte levels in HIV-1 infected individuals [80].

Andrographolide, neoandrographolide and 14-deoxy-11,12-didehydroandrographolide isolated from *A. paniculata *demonstrated viricidal activity against herpes simplex virus 1 (HSV-1) without significant cytotoxicity [81]. The *A. paniculata *ethanol extract and andrographolide inhibit the expression of Epstein-Barr virus (EBV) lytic proteins during the viral lytic cycle in P3HR1 cells, an oral lymphoma cell line latently infected by EBV. Andrographolide inhibits the production of mature viral particles and is not toxic to P3HR1 cells [82].

A recent *in vitro *study investigated the anti-influenza activity of *A. paniculata *in canine kidney cell line as well as mice infected with H1N1, H9N2 or H5N1 [83]. A newly synthesized andrographolide derivative 14-α-lipoyl andrographolide was more effective against avian influenza A (H9N2 and H5N1) and human influenza A H1N *in vitro *than andrographolide [83]. Another andrographolide analogue 14-glycinyl andrographolide hydrochloride inhibits virulence factor production and bacterial growth [84]. Moreover, a double blind, placebo-controlled, parallel-group clinical study on a combined formula of *A. paniculata *extract and *Acanthopanax senticocus*, also known as *Kan Jang*, demonstrated the formula's positive effects in treating acute upper respiratory tract infections and relieving the inflammatory symptoms of sinusitis [85].

The migratory response of leukocytes to chemokines forms the first line of defence to the invading microbial. *A. paniculata *extract inhibits secretion of RANTES, a potent chemoattractant exacerbating bronchial inflammation as a result of H1N1-infected human bronchial epithelial cells [86]. Andrograpanin enhanced chemokine stromal cell-derived factor-1α (SDF-1α) induced chemotaxis in Jurkat and THP-1 cells via CXC chemokine receptor-4 specific induced cell chemotaxis [87]. Andrograpanin enhancing chemokine-induced leukocyte chemotaxis may contribute to the anti-infectious function of *A. paniculata*. Post-translational cleavage by proprotein convertase is one of the several events that determine the viral infectivity and virulence [88]. The inhibitory action of andrographolide was enhanced significantly by the formation of dehydroandrographolide succinic acid monoester (DASM) *via *succinoylation [88]. DASM inhibits HIV by interfering with HIV-induced cell fusion and with HIV's binding to the cell [89].

#### Anti-hepatotoxic effects

Liver metabolizes xenobiotics, such as drugs, toxins and chemical carcinogens; chronic liver injury leads to cirrhosis. Anti-hepatotoxic enzymes include cytochrome P450 (CYP) super-family, or normalizing the levels of marker enzymes for the liver function test, such as glutamate oxaloacetate transaminase (GOT), glutamate pyruvate transaminase (GPT), acid phosphatase (ACP) and alkaline phosphatase (ALP) [90].

An early study showed that pre-treatment of mice with andrographolide, andrographiside and neoandrographolide alleviated hepatotoxicity induced by intoxication of carbon tetrachloride (CCl_4_) or tert-butylhydroperoxide (tBHP) in mice [91]. The glucoside groups in andrographolid and neoandrographolide were suggested to act as strong antioxidants. The *A. paniculata *aqueous extract and andrographolide decreased oxidative stress in isolated rat lymphocytes exposed to nicotine [92]. The *A. paniculata *aqueous extract and andrographolide ameliorated the dysfunction in the brain associated with nicotine toxicity [93]. Arabinogalactan, another aqueous component of the *A. paniculata*, Tris-buffer extract and andrographolide minimized the toxicity in pre-treated mice [90]. Oral treatment of rats with the *A. paniculata *methanol extract followed by CCl_4 _administration restored plasma lipid peroxidation, alanine transaminase (ALT) and aspartate transaminase (AST) [94].

Andrographolide significantly induced the expression of CYP1A1 and CYP1A2 mRNAs in a concentration-dependent manner, and synergistically induced CYP1A1 expression with the typical CYP1A inducers [95,96]. In addition, the *A. paniculata *60% ethanol extract or andrographolide may cause herb-drug interactions through CYP3A and CYP2C9 inhibition *in vitro *or CYP2C11 inhibition *in vivo *[97,98]. Induction of drug-metabolizing enzymes is considered to be an adaptive response to a cytotoxic environment. The *A. paniculata *ethanol extract, EtOAc extract and andrographolide induce the expression of the pi class of glutathione S-transferase, a phase II biotransformation enzymes involved in detoxification of various classes of environmental carcinogens, in rat primary hepatocytes [99]. A recent study showed that this induction by andrographolide was suppressed by the increase of cAMP or cAMP analogues [100].

#### Anti-atherosclerotic effects

Zhang *et al*. reported that the *A. paniculata *aqueous extract lowers systolic blood pressure (SBP) of both spontaneously hypertensive rats (SHR) and the control Wistar-Kyoto rats [101]. The *A. paniculata *water, n-butanol and aqueous extracts produce a significant fall in mean arterial blood pressure (MAP) without significant decrease in heart rate in anaesthetized Sprague-Dawley rats [102]. The 14-deoxyandrographolide isolated from the *A. paniculata *methanol extract showed vasorelaxant effects in isolated rat thoracic aorta [103]. Another diterpenoid isolated from *A. paniculata *methanol extract, 14-deoxy-11,12-didehydroandrographolide, significantly reduces MAP and heart rate and beating rate of isolated right atria in anaesthetised rats [104]. These two diterpenoids may exert their vasorelaxant activities through the activation of the NOS and guanylyl cyclase for NO release from endothelial cells [105]. Moreover, 14-deoxyandrographolide reduces the contractile response by calcium channel-dependent rat uterine smooth muscle contraction [106] The vascular smooth muscle is the major site of the hypotensive effects of the *A. paniculata *hot water extract and 14-deoxy-11,12-didehydroandrographolide [107], suggesting relaxant effects of *A. paniculata *in muscle.

Andrographolide suppresess apoptosis of human umbilical vein endothelial cells (HUVECs) induced by growth factor deprivation via the activation of PI3/Akt pathway [108]. The aqueous extracts significantly decreased platelet aggregation *in vitro *[107]. Andrographolide and 14-deoxy-11,12-didehydroandrographolide significantly inhibited thrombin-induced platelet aggregation whereas neoandrographolide had little or no activity. The inhibition of ERK1/2 pathway may contribute to anti-platelet activity [109]. Four flavonoids, namely 7-*O*-methylwogonin, apigenin, onysilin and 3,4-dicaffeoylquinic acid inhibit collagen, arachidonic acid, thrombin and platelet activation factor induced platelet aggregation; 14-deoxy-11,12-dihydroandrographolide demonstrated moderate vasorelaxing effects in isolated rat thoracic aorta [110].

#### Anti-hyperglycaemic effects

Hyperglycaemia is involved in the aetiology of development of diabetic complications. Hypoglycaemic herbs increase insulin secretion, enhance glucose uptake by adipose or muscle tissues and inhibit glucose absorption from intestine and glucose production from liver [111]. Oral administration of the *A. paniculata *ethanol extract significantly reduced the fasting serum glucose level in streptozotocin (STZ) induced diabetic rats. No significant change in insulin level was observed among the three groups of diabetic rats. The activity of hepatic glucose-6-phosphalase (G-6-Pase) and fasting serum triglyceride levels were significantly reduced by the *A. paniculata *extract [112]. In addition to its hypoglycaemic action, the *A. paniculata *may also reduce oxidative stress in diabetic rats [113]. An *in vitro *study and *in vivo *oral carbohydrate tolerance tests in STZ-induced diabetic rats suggest that α-glucosidase inhibition may be responsible for the anti-diabetic activity of *A. paniculata *ethanol extract [114].

The *A. paniculata *aqueous extract significantly reduces blood glucose in hyperglycaemic rats without significantly changing the rats' weight [115]. However, alloxan-induced diabetic rats treated with *A. paniculata *water extract had higher body weight than the positive (diabetic) controls; the blood glucose levels were significantly reduced and impaired oestrous cycle in alloxan-induced diabetic rats was restored [116].

Andrographolide significantly attenuated the increase of plasma glucose induced by an intravenous glucose challenge test in normal rats. Andrographolide enhanced the uptake of glucose and the mRNA and protein levels of the glucose transporter subtype 4 (GLUT4) in soleus muscle in STZ-diabetic rats [117]. Andrographolide not only reduced expression of phosphoenolpyruvate carboxykinase (PEPCK) in liver of STZ-diabetic rats, activated α1-adrenoceptors to enhance the secretion of β-endorphin, thereby stimulating the opioid μ-receptors to reduce hepatic gluconeogenesis and to enhance the glucose uptake in soleus muscle, leading to a decrease of plasma glucose in STZ-diabetic rats [118]. A recent study showed that andrographolide-lipoic acid conjugate (an andrographolide analogue) had both hypoglycaemic and beta cell protective effects [119].

#### Anti-oxidative activities

Antioxidant action is manifested by a decrease of malondialdehyde (MDA) formation via lipid peroxidation and an increase of hepatic antioxidative enzymes and antioxidants such as glutathione peroxidase (GPX), glutathione reductase (GR), catalase (CAT), superoxide dismutase (SOD) and glutathione S transferase (GST). Anti-oxidative activity of *A. paniculata *contributes to its anti-inflammatory, anti-cancer, anti-hepatotoxic, anti-atherosclerotic and anti-diabetic activities [27,44,91,108,117].

An *in vitro *scavenging of superoxide radical assay suggests that neoandrographolide from the *A. paniculata *hexane/EtOA_C _extract is an effective *in vivo *scavenger for small radicals [120].

An *in vivo *study demonstrated that the *A. paniculata *80% ethanol extract enhanced murine hepatic antioxidative enzymes and antioxidants such as GPX, GR, CAT and SOD but reduced lipid peroxidation [121]. The *A. paniculata *methanol extract significantly lowered MDA levels and raised the total antioxidant status in urine samples 24 hours after oral administration [122]. The *A. paniculata *methanol extract preserved CAT and SOD activities in erythrocytes after CCl_4 _administration [94]. Oral administration of the *A. paniculata *aqueous extract significantly enhanced CAT, SOD and GST activities in the liver of lymphoma bearing mice [123]. Moreover, the *A. paniculata *aqueous extract exhibited more antioxidant action than its ethanol extract in terms of free radical scavenging, xanthine oxidase inhibition and anti-lipid peroxidation [124].

## Conclusion

Among the single compounds extracted from *A. paniculata*, andrographolide is the major one in terms of bioactive properties and abundance. Among the andrographolide analogues, 14-deoxy-11,12-didehydroandrographolide is immunostimulatory, anti-infective and anti-atherosclerotic; neoandrographolide is anti-inflammatory, anti-infective and anti-hepatotoxic; 14-deoxyandrographolide is immunomodulatory and anti-atherosclerotic. Among the less abundant compounds from *A. paniculata*, andrograpanin is both anti-inflammatory and anti-infective; 14-deoxy-14,15-dehydroandrographolide is anti-inflammatory; isoandrographolide, 3,19-isopropylideneandrographolide and 14-acetylandrographolide are tumor suppressive; arabinogalactan proteins are anti-hepatotoxic. The four flavonoids from *A. paniculata*, namely 7-*O*-methylwogonin, apigenin, onysilin and 3,4-dicaffeoylquinic acid are anti-atherosclerotic.

## Abbreviations

TNF-α: tumour necrosis factor-α; IL-1: interleukin-1; IFN-γ: interferon-γ; NO: nitric oxide; ERK1/2: extracellular signal-regulated kinase1/2; MAPK: mitogen-activated protein kinase; JNK: c-Jun NH_2_-terminal kinase; VEGF: vascular endothelial growth factor; TIMP-1: tissue inhibitors of metalloproteinase-1; MMP-7: matrix metalloproteinases-7; hPBL: human peripheral blood lymphocytes; NFAT: nuclear factor of activated T cells; EAE: experimental autoimmune encephalomyelitis; BALF: bronchoalveolar lavage fluid; HIV: human immunodeficiency virus; HSV-1: herpes simplex virus 1; EBV: Epstein-Barr virus; SDF-1α: stromal cell-derived factor-1α; CYP: cytochrome P450; CCl_4_: carbon tetrachloride; tBHP: rert-butylhydroperoxide; ALT: alanine transaminase; AST: aspartate transaminase; SBP: systolic blood pressure; SHR: spontaneously hypertensive rats; HUVECs: human umbilical vein endothelial cells; STZ: streptozotocin; GLUT4: glucose transporter subtype 4; MDA: malondialdehyde; GPX: glutathione peroxidise; GR: glutathione reductase; CAT: catalase; SOD: superoxide dismutase; GST: glutathione S transferase

## Competing interests

The authors declare that they have no competing interests.

## Authors' contributions

BFL and WWC searched the literature and drafted the manuscript. All authors read and approved the final version of the manuscript.
